# Factors influencing the removal torque of palatal implant used for orthodontic anchorage

**DOI:** 10.1186/s40510-021-00359-3

**Published:** 2021-06-07

**Authors:** Marc Andreas Schätzle, Monika Hersberger-Zurfluh, Raphael Patcas

**Affiliations:** Clinic of Orthodontics and Pediatric Dentistry, Center of Dental Medicine, University of Zurich, Plattenstrasse 11, CH-8032 Zurich, Switzerland

**Keywords:** Skeletal anchorage, Removal, Complications, Surgical, Palatal implants, Human, Torque

## Abstract

**Background:**

A non-invasive method has recently been introduced to remove osseo-integrated palatal implants by using the implantation ratchet which is designed to screw in or unscrew the implants. Although a proof of concept has been published, the torque involved to successfully explant have not been investigated so far. The aim of this study was to assess the removal torque required to explant osseo-integrated and orthodontically utilized palatal implants, and to identify potentially influencing variables.

**Materials and method:**

Thirty-one consecutive patients (15 females, 16 males; mean age 24.1 ±7.4 years) with fully osseo-integrated and previously orthodontically loaded palatal implants (Orthosystem®: diameter 4.1mm/length 4.2mm/sandblasted with large grits (SLA) surface) were randomly assigned to either clockwise or counter-clockwise non-invasive explantation. The respective explantation tool with an electric torque control was placed on the abutment connection of the implant and secured by an occlusal screw.

The primary outcome studied was maximal removal torque (MRT) needed to detach the implant from its socket which was recorded individually together with other potentially influencing secondary outcomes (gender, age, orthodontic loading time, use of local anaesthetics). Student’s *t*-test was used to contrast MRT difference for the gender, type of suprastructure, use of local anaesthetics, and rotational direction.

Spearman correlations was used to investigate associations between MRT and patient’s age or duration loading time.

**Results:**

Average MRT (148.6 ± 63.2N/cm) using ratchet as a non-invasive removal method of palatal implant was considered safe. The triangular head fractured of palatal implant at a torque level of 300.1 Ncm. Significantly higher explantation were recorded for male patients compared to female patients (182.0 ± 63.0 Ncm vs 112.8 ± 40.8 Ncm; P=0.001). On the other side, the mean removal torque for palatal removal in clockwise direction was non-significantly different (158.3 ± 58.6 Ncm) compared to counter-clockwise direction (139.4 ± 67.9 Ncm). Neither patient’s age (*p*=0.324) nor loading time (*p*=0.214) were significantly correlated with removal torque values.

**Conclusions:**

Pertinent literature on this subject is practically non-existent, as orthodontics is presumably the only discipline where implant removal represents a treatment success. Mean MRT for successful palatal implant removal was 148.6±63.2Ncm, but a large spectrum was observed (minimum 31.5Ncm, maximum 272.8Ncm). This obvious heterogeneity underlines the importance to investigate possible influencing factors. The safe and simple non-invasive method for palatal implant removal necessitates moderate, but not high torque MRTs, independently of the torque direction. The necessary MRT seems clearly influenced by gender, but less so by patient’s age or loading time.

## Introduction

Reliable anchorage is required in various orthodontic treatment approaches to achieve satisfactory result. The most common appliance to achieve anchorage reinforcement is a headgear, depending on a high degree on patient’s cooperation [9, 15]. Temporary anchorage devices (TAD) were introduced [4, 10, 21, 22] and offer reliable and predictable skeletal anchorage for orthodontic treatment. Comparing different TADs, it has been shown that rough surfaced palatal implants and miniplates have a statistically significant higher survival rate than miniscrews [17].

Short, rough-surfaced palatal implants are an established and reliable approach to reinforce orthodontic anchorage [13, 17, 25]. They are clinically satisfactory when their entry point into the cortical bone is at between the anterior-posterior level of the maxillary first and second premolars—perpendicular to the palatal surface [13]. Until recently, its removal was only possible surgically using a hollow cylinder trephine. This standard method removes the implant together with a larger bone volume and is therefore considered invasive and is not free of complications [5]. Lately, an explantation tool has been developed which allows a sufficient torque application to break the bone-implant-interface, thereby enabling to simply unscrew the palatal implant [8].

Due to its minimal invasiveness, use of this new custom explantation tool seems to be associated with significantly less adverse patient reaction and medical complications than the surgical removal by the respective trephine. Furthermore, this new explantation method is technically easier and better tolerated by patients [5].

One of the reported complications with the new removal method was fracture of the top triangular implant part (1mm), leaving the rest of the implant still in place [12]. The reasons for this breakage could be either an improper placement of the removal tool, an insufficient initial loosening, thereby concentrating all the torque on the abutment connection, a denser bone requiring a higher removal torque value, or a material weakness of the implant. It should be noted that in the respective failure case subsequent removal with the traditional method by trephine was still possible without any occurring problems. The novel non-invasive method presents obvious benefits, including a less technique-sensitive implant removal, but introduces new questions that must be addressed. Choosing to remove an osseo-integrated implant by unscrewing rather than trepan drilling requires to establish the torque needed and calls for a careful analysis of variables that might potentially influence a successful outcome.

The purpose of the present study was therefore to assess the maximal removal torque recorded at removal by for unscrewing successfully osseo-intergrated and orthodontically loaded palatal implants by contrasting gender, type of suprastructure, use of local anaesthetics, and rotational direction. The second objective was to investigate the possible influence of correlation of maximal removal torque (MRT) with age and duration of loading.

## Material and methods

Thirty-four consecutive patients with successfully osseointegrated orthodontic palatal implant (16 females, 18 males) were recruited for the prospective study from a private practice (MS, Lucerne, N =27) and from the Clinic of Orthodontics and Pediatric Dentistry, Centre of Dental Medicine, University of Zurich, Zurich, Switzerland (N=7). All patients had previously received a second-generation Orthosystem® palatal implant for anchorage reinforcement during orthodontic treatment.

The palatal implants were made of pure titanium with a diameter of 4.1mm, an endosteal part with SLA (sandblasted with large grits of 0.25–0.5 mm and acid etched with HCl/H2SO4) surface of 4.2-mm length, a smooth neck of 1.8-mm height, and an almost triangular-shaped abutment connection of 1-mm height (Orthosystem®, Institute Straumann AG, Basel, Switzerland).

Subjective experience reported by earlier patients indicated that the explantation might be possible without local anaesthesia as patients feel only momentary pressure during the initial loosening [12]. It was therefore left to the patient’s discretion whether the explantation was performed under local anaesthesia or not (apart for implants that were covered by gingiva, were local anaesthesia was non-negotiable).

**Fig. 1:**
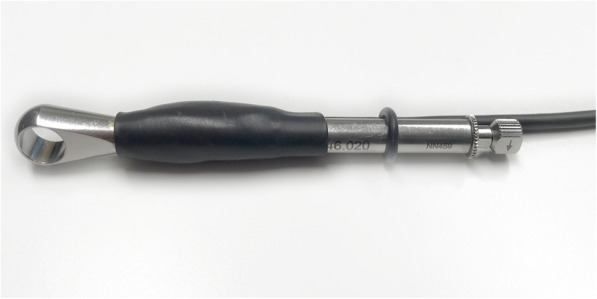
Electric torque control ratchet

Prior to the start of the study, all enrolled patients were randomly assigned to either clockwise or counter-clockwise non-invasive explantation. Explantation was performed by means of the ratchet usually dedicated for implant insertion. For the purpose of this study, a calibrated electric torque control was integrated in the ratchet (Fig. 1). The explantation tool [8] was placed on the 1-mm high triangular abutment connection of the implant and secured by an occlusal screw made of hardened steel, similar to the fixation of a normal prosthodontic implant abutment. Only after a tight and gapless fit has been ensured, the ratchet with an electric torque control was fixed on the respective explantation tool, ensuring that the ratchet was not tilted in any direction at all.

The maximal removal torque (MRT) needed to detach the implant from its bony socket was recorded individually, together with unscrewing direction and all other potentially influencing variables (gender, age, type of suprastructure[active; passive] and duration of orthodontic load).

Ethical guidelines [23] and State and Federal Law (Human Research Act SR 810.30) were strictly followed, and a waiver was obtained by the local governmental ethics committee prior to the study (BASEC Nr. Req-. 2018-00561). Written informed consent, according to the directives stipulated by the governmental ethics committee, was obtained from each patient, before the start of the study.

Of the 34 consecutive patients with successfully osseointegrated palatal implants assigned for implant removal, three were excluded from statistical analysis for various reasons: implant was not orthodontically loaded (*n*=1), measurement error (*n*=1), and patient at high risk (*n*=1).

Statistical analysis was performed with SPSS (Version 25, IBM Corp., Armonk, NY, USA). After checking for normal distribution with the Kolmogorov-Smirnov test, descriptive statistics were calculated including means and standard deviations (SD). Differences between the groups were investigated with Student’s *t*-tests, and wherever applicable a Spearman-Rho test or a Pearson correlation test were conducted to identify possible associations between torque and age or loading time, respectively. Significance was set at two-tailed alpha ≤ 0.05.

## Results

The description of the participants and the assessed factors are presented in Table 1. The average time of orthodontic loading was 3.4 ± 0.9 years. A total of 17 (54.8%) palatal implants had been used for active tooth movement, whereas 14 (45.2%) for passive reinforcement of anchorage segments. Overall, 22 (71%) non-invasive explantations were performed without any anaesthesia.

**Table 1:** Subject characteristics

	*N*	Years	%
**Participants**	31		
Sex			
Female	15		48
Male	16		52
Age at explantation		23.2 ± 6.5	
**Palatal implant specifications**			
Type of suprastructure			
Passive	14		45
Distalizer	8		26
Mesializer	9		29
Duration of loading		3.4 ± 0.9	
Use of local anaesthetics			
Yes	9		29
No	22		71
Rotational direction			
Clockwise	15		48
Anti-Clockwise	16		52

Implant removal with the new non-invasive method was completely successful in all but one (96.8 %) patient with a recorded MRT value for successful explanation of 148.6 Ncm (SD=63.2. Ncm) (Table 1). In one case, there was a fracture at the abutment connection (1 mm) of the palatal implant, leaving the remaining part of the implant still in situ. Since the implant body was unharmed, subsequent removal with the traditional method by trephine was still possible without difficulties. The triangular head fractured at a torque level of 300.1 Ncm.

**Fig. 2:**
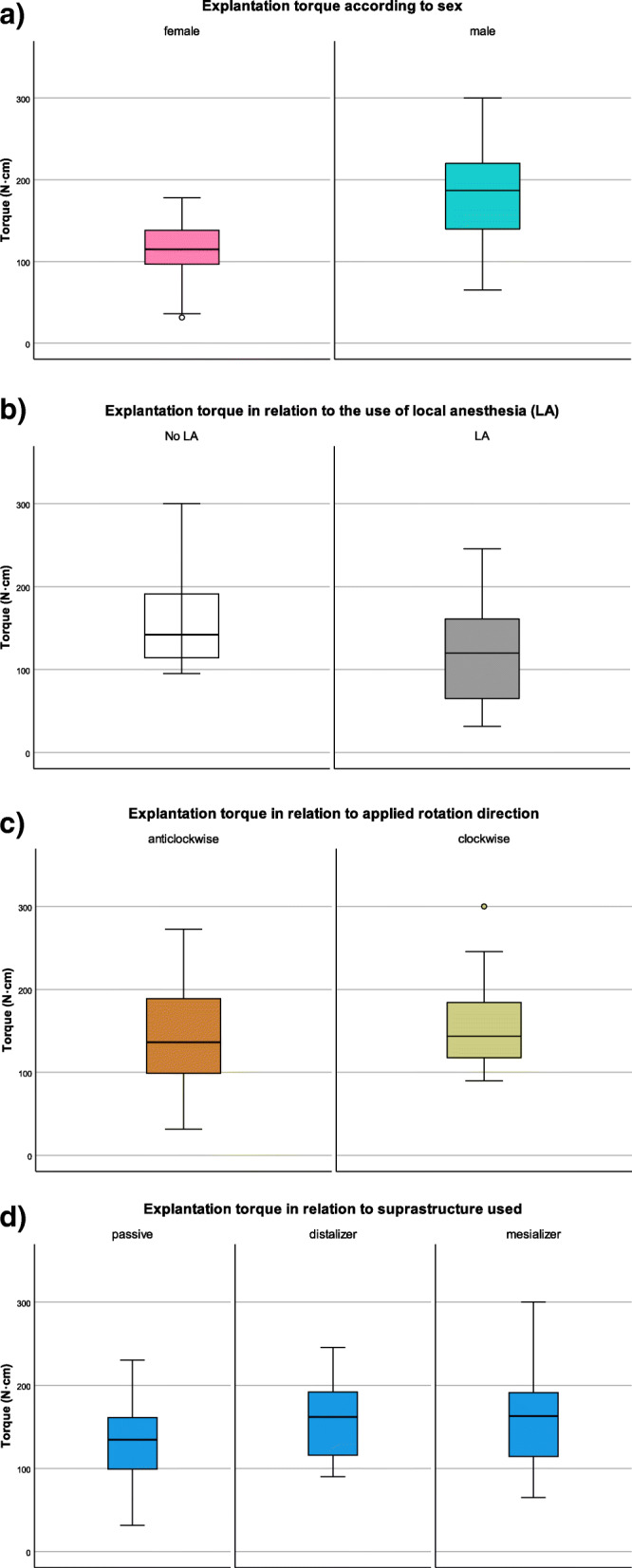
Box and whisker plot of the explantation torque needed depending on sex (**a**), use of local anaesthesia (**b**), applied rotation direction (**c**), and the suprastructure previously used (**d**)

Normal distribution was ascertained for the variables torque (Kolmogorov-Smirnov *P*=0.20) and duration of loading (Kolmogorov-Smirnov *P*=0.21), but not for patient’s age (Kolmogorov-Smirnov *P*=0.001). The statistical analysis indicated that significantly higher MRT needed to be applied in male patients (182.0±63.0 Ncm) compared to female patients (112.8±40.8Ncm) to overcome the bone-implant interface (*P*=0.001). The modus of suprastructure loading did not seem to be significantly associated with measured MRT (*p*=0.19), but implants that were previously actively loaded tended to require higher removal torque (162±16.1 Ncm) compared to passively loaded ones (140.0±15.3 Ncm) (Fig. 2a–d, Table 2). The mean applied MRT for clockwise explantation was only slightly (13%) higher than the anti-clockwise, unwinding explantation direction, which was not significantly different. Correlation coefficients and *p*-values for possible associations between the variables, based on Pearson’s *r* tests for the variables “Explantation torque” and “Duration of Loading”, and Spearman-Rho tests for the variable “Age”, are depicted in Table 3.

**Table 2:** *T*-test results for comparison of the various palatal implant variables

	***n***	Mean torque (Ncm ± SD)	***p*** value
**Mean MRT**	31	148.6 ± 63.2	
**Sex**			
Female	15	112.8 ± 40.8	0.001*
Male	16	182.0 ± 63.0	
**Type of suprastructure**			
Passive	14	140.0 ± 15.3	0.19
Active (mesializer or distalizer)	17	162.2 ± 16.1	
**Use of local anaesthetics**			
Yes	9	121.1 ± 72.9	0.18
No	22	160.0 ± 56.8	
**Rotational direction** ***n***			
Clockwise	15	158.3 ± 58.6	0.41
Anti-clockwise	16	139.4 ± 67.9

**Table 3:** Table of correlation

		MRT (N cm)	Duration of loading (d)	Age (y)	Use of local anaesthesia	Type of suprastructure	Rotational direction
MRT(N cm)	Corr. Coeffic.		−0.230	0.183	−0.283	0.232	0.152
	*Significance*		*0.214*	*0.324*	*0.124*	*0.209*	*0.414*
Duration of loading (d)	Corr. Coeffic.	−0.230		−0.71	0.087	−0.259	−0.130
	*Significance*	*0.214*		*0.704*	*0.641*	*0.159*	*0.484*
Age (y)	Corr. Coeffic.	0.183	−0.71		−0.207	−0.038	−0.123
	*Significance*	*0.324*	*0.704*		*0.265*	*0.840*	*0.511*

Correlation coefficients and *p*-values for possible associations between the variables, based on Pearson’s *r* tests for the variables “Explantation torque” and “Duration of Loading”, and Spearman-Rho tests for the variable “Age”

**Fig. 3:**
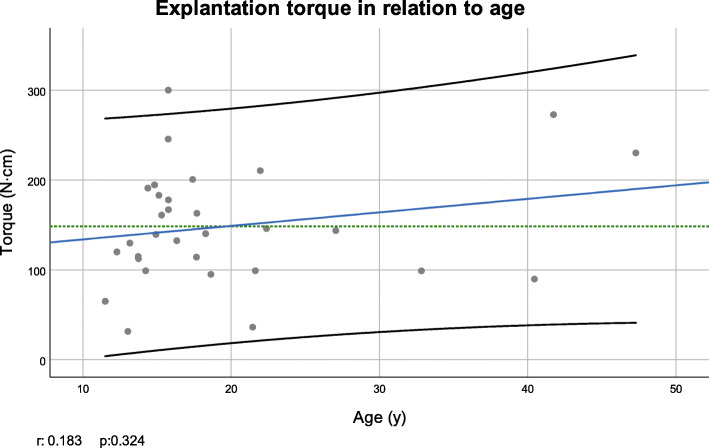
Scatter plot and Spearman correlation coefficient (*r*) of torque value and age. Green dotted line: mean torque; blue line: linear regression curve; black lines: 95% confidence interval

**Fig. 4:**
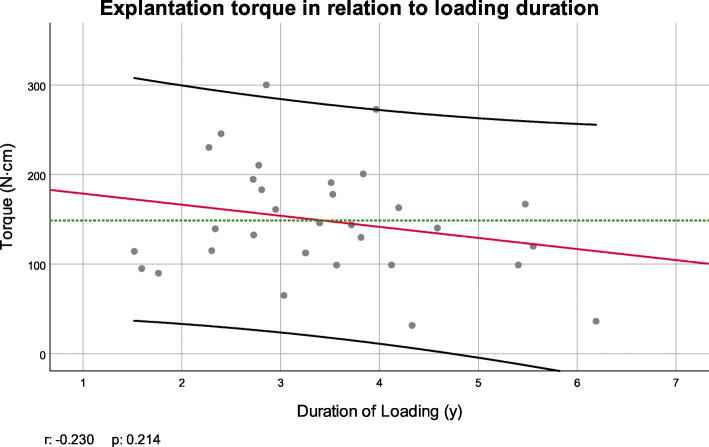
Scatter plot and Pearson correlation coefficient (*r*) of torque value and duration of loading. Green dotted line: mean torque; red line: linear regression curve; black lines: 95% confidence interval

Recorded MRT values according to patient age are given in a scatter plot including a fitted linear trend order to disclose a possible relationship between MRTe and patient’s age; a scatter-diagram was plotted, including a linear regression line and its 95% confidence interval (Fig. 3). Age did not follow normal distribution (Kolmogorov-Smirnov test *p* < 0.001). Spearman-Rho test revealed no significant correlation between the MRT needed for explantation and age (*p* = 0.324). Similarly, the Scatter-diagram plotting recorded MRT on loading duration is given in Fig. 4. Here, a trend towards smaller required torque values in patients with longer loading duration could be observed, which again was not statistically significant (Pearson test *p*=0.214).

## Discussion

Since the introduction of bone-borne temporary anchorage devices (TADs), orthodontists are no longer solely dependent on non-predictable mechanics that necessitate the patient’s often insufficient cooperation [15]. Of all introduced TADs, orthodontic palatal implants like the Orthosystem® (Institut Straumann AG; Basel, Switzerland) are more capable of providing reliable absolute orthodontic anchorage owing to their osseointegration/low failure rate and are therefore considered to be superior to other orthodontic tooth-borne anchorage devices [17]. Yet a major downside of palatal implants was that their removal was only possible surgically using a hollow cylinder trephine. This standard method removes the implant together with a larger bone volume and is therefore considered invasive and is not free of known complications [5]. More recently, an explantation tool has been developed which allows a sufficient torque application to break the bone-implant-interface, thereby enabling to simply unscrew the palatal implant [8].

This paradigm shift in removal of osseointegrated palatal implants (i.e. unscrewing rather than trepanning) necessitates to establish an evidence base concerning the required unscrewing torque. The pertinent literature on this subject is practically non-existent, as orthodontics is presumably the only discipline where intact implant removal is seen as a treatment success. Few investigations exist for prosthodontic implant removal [1, 6, 16, 19] or miniscrew removal (e.g. [2, 3, 11, 18, 20]). All these scarce studies remain of little value for comparison, because their differences to the present investigation are too important to be ignored: different loading (prosthodontic vs. orthodontic), region of insertion, TAD dimensions in length, width and overall design, and surface characteristics differ significantly one from each other and disqualify any direct comparison.

However, Favero et al. [6] explanted 8 small screw titanium implants (Exacta MS series conical profile, with a diameter of 3.3 mm and a length of 7.0 mm) used as indirect orthodontic anchorage inserted in the palate. A large discrepancy between the torque values obtained in vitro (which are similar to ours) and in-vivo was reported. The small numbers and this uncertainty warrant caution for any attempt to generalize their observation. Our study’s strength is not only the larger sample size which makes generalizing more acceptable, but also its novel insights gained by discriminating and analysing statistically co-variates, such as type of suprastructure, duration of loading, use of anaesthesia, age and sex. Lastly, in contrast to the present findings, Favero and colleagues reported breakage of the implant at a significantly lower torque level of ca. 210Ncm.

A study analysing the MRT of sandblasted sand-blasted, large-grit, and acid-etched surface-treated mini-implant (C-implant, CImplant Co, Seoul, South Korea) [11] (1.8 mm in diameter and 8.5 mm in length) showed, however, no fracture or distortion during removal, and the mean MRT was 16.4 Ncm (range, 3.94–35.41 N per centimeter).

Implant removal with the new non-invasive method was completely successful in all but one (96.8 %) patient. Since in this single case the triangular head fractured at a very high-MRT level of 300.1 Ncm, the use of a torque control gauge might be advantageous to avoid implant head fracture. The mean MRT of successful palatal implant removal was 148.6±63.2Ncm, but it must be stressed that a large spectrum was observed (minimum 31.5Ncm, maximum 272.8Ncm). This obvious heterogeneity underlines the importance to investigate possible influencing factors.

To the best of our knowledge, only one prospective study on prosthetic implant removal has been published. Explanation reasons were periimplantitis, fracture, or malpositioning [1]. Explantation was performed by application of counter-clockwise torque using a dedicated extraction kit (BTI Biotechnology Institute, Vitoria, Spain) to break the implant–bone union. Only in cases where the torque wrench opened (the maximum of wrench torque was set at 200 Ncm), the extraction assembly was removed and specialized trephine burs were employed to cut into the first 3 to 4 mm of implant-bone contact. The mean removal torque for explantations of implants without using the trephine method was 146 ± 5 Ncm. Well aware of the due caution that must be exercised when comparing this study to our results, it is nevertheless of interest to note that the mean unscrewing MRT were very similar. Anitua and colleagues failed to present data on the dimensions of the explanted implants (length, diameter), gender, or loading time, all this further restricts the validity of the comparison. Probably of modest scientific value is the report of three implants removed in a human volunteer by reverse torque to failure at torque levels between 45 and 58 Ncm [19], which is in the lower range of the present data.

Our results clearly demonstrate that unscrewing MRT were significantly higher in male (182.0±63.0 Ncm) than in female patients (112.8±40.8Ncm). It is probably not erroneous to assume that this disparity is associated to the gender variation known in palatal bone thickness and density [24].

Perhaps somewhat surprisingly, the type of suprastructure loading (active mesializer or distalizer versus passive loading) did not have any significant effect on the measured MRT. Yet a certain trend in the data can be discerned: Implants actively loaded tended to necessitate higher MRT. It is fair to assume that the underlying reason might be associated to a higher degree of osseointegration which might have evolved during active loading. This observed tendency seems to be in accordance with previous investigations indicating that bone tissue turnover as well as the density of the alveolar bone is higher adjacent to loaded implants compared to unloaded implants [14] and might thus confirm the hypothesis posted by Frost which stipulates that mechanical agents adopt an important role in bone metabolism [7]. The importance of bone metabolism is probably mirrored in the scatterplot visualizing the explantation torque in relation to age. Although no correlation between MRT and age could be disclosed, a certain trend was evident. Here too, an association between bone density (i.e. greater brittleness with age) and higher unscrewing torque might be speculated. Of no discernible impact on explantation torque were the use of local anaesthetics and the removal direction (clockwise versus anti-clockwise). The latter demands interpretation. Assuming successful osseointegration, unscrewing the implant causes a breakage, either at the implant-bone interface or fully within the adjacent bone. Histological investigation on the exact anatomical region of breakage would certainly help to understand why rotational direction does seemingly not affect the required MRT.

## Conclusions

- The application of shear stress at the implant-bone contact was effective to permit atraumatic palatal implant explanation.
- It necessitates moderate, but not high MRT, independently of the torque direction. The necessary MRT seems clearly influenced by gender, but less so by patient’s age or loading time.
- The introduction of a torque control gauge might be necessary to avoid implant head fracture in the future.

## Data Availability

The datasets used and/or analysed during the current study are available from the corresponding author on reasonable request.

## Abbreviations

MRT: Maximum removal torque
SD: Standard deviations
SLA: Sandblasted with large grits of 0.25–0.5 mm and acid etched with HCl/H2SO4
TAD: Temporary anchorage devices
